# Stem Cell Therapy for Sequestration of Traumatic Brain Injury-Induced Inflammation

**DOI:** 10.3390/ijms231810286

**Published:** 2022-09-07

**Authors:** Mia C. Borlongan, Susanna Rosi

**Affiliations:** 1 Department of Physical Therapy and Rehabilitation Science, University of California, San Francisco, CA 94143, USA; 2 Brain and Spinal Injury Center, University of California, San Francisco, CA 94110, USA; 3 Department of Neurological Surgery, University of California, San Francisco, CA 94143, USA; 4 Weill Institute for Neuroscience, University of California, San Francisco, CA 94158, USA

**Keywords:** trauma, traumatic brain injury, stem cell-based therapy, resident stem cells, neurogenesis, inflammation, brain repair

## Abstract

Traumatic brain injury (TBI) is one of the leading causes of long-term neurological disabilities in the world. TBI is a signature disease for soldiers and veterans, but also affects civilians, including adults and children. Following TBI, the brain resident and immune cells turn into a “reactive” state, characterized by the production of inflammatory mediators that contribute to the development of cognitive deficits. Other injuries to the brain, including radiation exposure, may trigger TBI-like pathology, characterized by inflammation. Currently there are no treatments to prevent or reverse the deleterious consequences of brain trauma. The recognition that TBI predisposes stem cell alterations suggests that stem cell-based therapies stand as a potential treatment for TBI. Here, we discuss the inflamed brain after TBI and radiation injury. We further review the status of stem cells in the inflamed brain and the applications of cell therapy in sequestering inflammation in TBI.

## 1. Traumatic Brain Injury and Inflammation

Traumatic brain injury (TBI) is a growing health problem due to the chronic behavioral and cognitive impairments that affect the quality of life of millions of individuals [1,2,3,4,5]. Its incidence is between 1.5–3.8 million people, who succumb to TBI each year in the United States alone [6,7]. In terms of age-specific TBI incidence, Americans aged 0 to 4 years show the highest rate of TBI-related emergency department visits (about 1200 per 100,000 population), then those aged 15 to 19 years (about 750 per 100,000), and the most prone to hospitalizations (about 340 per 100,000) and deaths (about 60 per 100,000) are those aged 75 years and older [8,9,10,11]. For sex-specific TBI incidence, men appear to suffer TBI about 40% more than women in the general adult population, but this sex difference diminishes at age 75 years and older [12,13]. Mortality rate reveals that TBI accounts for about 30% of all injury-related deaths in the United States [12,13]. Severity classification of TBI has largely categorized the injury into three types, namely, mild, moderate, and severe, based on the normal, partially abnormal, and fully abnormal structural appearance of the skull, respectively, with corresponding loss of consciousness at less than 30 min, between 30 min and 24 h, and more than 24 h following the initial injurious episode [14]. TBI prognosis is challenging because of disease heterogeneity, but Glasgow Coma Scale motor score, age, and pupillary activity appear to serve as good prognostic factors [15]. Similarly, TBI treatments vary, with each case approached differently, based on the severity of the injury, which may include non-invasive modalities, such as drug administration, and invasive surgery, including bilateral decompressive craniectomies [16]. Moreover, while TBI has been traditionally considered to be an acute injury, survivors manifest many pathological features and symptoms reminiscent of neurodegenerative disorders, most notable Alzheimer’s disease. Indeed, there is an increased incidence of Alzheimer-like dementia after TBI, especially in aged patients [17,18]. Although the common definition of TBI entails a mechanical injury to the skull and brain tissue, a mild form of TBI that manifests without visible physical deformation of the skull has been well documented. Indeed, mild TBI is much more rampant in our soldiers and veterans [19,20,21]. Long-term symptoms of mild TBI may include chronic traumatic encephalopathy [22], Alzheimer’s disease [23], post-traumatic stress syndrome [24], and a realm of many symptoms of neurodegenerative disorders [25]. Multiple cell death mechanisms have been implicated in TBI, but a common pathologic manifestation involves aberrant inflammation [26,27,28]. Imaging and cytokine plasma, or cerebrospinal fluid (CSF) profiling, in both mild TBI patients [29,30,31] and animal models [16,17,18] reveal upregulated inflammation. The widely accepted TBI biomarkers employ inflammation-based assays, including glial fibrillary acidic protein, ubiquitin C-terminal hydrolase-L1, and C-reactive protein [24,25,26,27,28,29,30,31,32,33,34]. Reactive microglia and infiltration of peripherally derived macrophages accompany the disease progression of TBI, especially in the aged brain [35,36,37,38,39]. More specifically, our group demonstrated that peripherally-derived macrophages (CCR2^+^) propagate to the injured brain and participate in chronic TBI-induced cognitive deficits in young animals [35,38]. Using CX3CR1(GFP/+)CCR2(RFP/+) reporter mice, we detected that TBI triggered an increase in peripherally derived CCR2(+) macrophages within the hippocampus, a well-established brain area responsible for learning and memory [35]. Moreover, we dissected the key contribution of CCR2(+) macrophages to the resulting neuroinflammation in response to TBI [35]. Finally, we showed that targeting CCR2(+) macrophages with CCX872, a robust CCR2 selective antagonist, dampened the aberrant proliferation of inflammatory macrophages after TBI [35]. Altogether, we advanced a macrophage-directed therapy that effectively blocked CCR2 and attenuated TBI-induced inflammation, coincident with amelioration of hippocampus-associated cognitive deficits [35]. Importantly, in the aged brain we measured a remarkably higher number of peripherally-derived macrophages (i.e., monocytes) after TBI than in young, injured animals [38]. In our subsequent study, we examined the effects of aging on the crosstalk between sub-chronic response to TBI of peripherally-derived monocytes (CD45^hi^; CCR2^+^) and the onset of chronic cognitive deficits. We found that TBI aged mice displayed more upregulation of peripherally-derived monocytes than the TBI young animals [38]. Such elevated peripherally-derived monocytes positively correlated with enhanced CCR2 expression [38]. In tandem, the TBI aged animals exhibited more dysregulated myeloid cell populations coupled with deficient anti-inflammatory responses than the TBI young animals. In the circulation, blood CCR2^+^ monocyte population was increased in TBI aged animals but not in TBI young animals. Lastly, genetic depletion of CCR2 suppressed the infiltration of peripherally-derived monocytes and blocked chronic TBI-induced spatial memory deficits in TBI aged animals [38]. These two studies highlight that the surge of CCR2^+^ peripherally-derived macrophages is coupled with increased levels of CCL2 chemotactic ligands and upregulated blood CCR2^+^ monocyte population, likely contributing to the impaired inflammatory responses that plagued the TBI aged animals much more than the TBI young animals [22,25]. Interestingly, the aberrant infiltration of CCR2^+^ peripherally-derived macrophages measured up to seven days post TBI in aged animals could be sequestered by knocking out CCR2 leading to attenuation of spatial memory deficits [35,38]. These findings implicate the critical involvement of resident microglia and peripherally derived macrophages in TBI pathology and treatment [35,38].

The cellular and molecular changes that lead to chronic cognitive deficits in response to inflammation are associated, at least in part, with the complement initiation components C1q, C3, and CR3, which are known to regulate microglia-synapse interactions [39]. Both genetic and pharmacological blockade of the complement pathway prevented memory deficits in aged, injured animals [39]. Altogether, these preclinical studies represent some of the compelling preclinical evidence supporting the key role of inflammation in TBI-induced long term cognitive deficits in rodents.

## 2. Inflammation-Plagued TBI-like Events

Recognizing that TBI manifests an aberrant inflammation provides the impetus to consider subtle events that at first glance appear harmless but which can result in a detrimental condition reminiscent of TBI. Accumulating evidence points to uncontrolled radiation exposure arising from radiological terrorism, industrial accidents or military circumstances poses a serious threat for civilians [40,41,42]. Interestingly, irradiation increased the vulnerability of animals to exhibit cognitive deficits when subsequently exposed to TBI [40]. Along this line, although irradiation affords therapeutic effects against brain tumors, this treatment also induces long-term persistent cognitive impairments that are dependent on inflammatory cells [41,42,43]. Similar to what is observed in rodents after TBI [35] CCR2 deficiency prevents neuronal dysfunction and cognitive deficits after exposure to therapeutic brain irradiation [31] and radiation and TBI combined injury [40,44].

Moreover, the use of whole-body irradiation to facilitate bone marrow engraftment leads to long-term and brain-wide cognitive and synaptic deficits [45]. Indeed, cognizant of the gamut of stressors that accompany deep space travels, (including ionizing radiation, gravitational changes during flight and in orbit, psychological stress from a confined environment and social isolation) galactic cosmic radiation exposure represents the most dangerous of stressors [46,47]. Each deep spaceflight stressor, or a combination thereof, may represent unique circulating immune responses coupled with an altered immune system, as evidenced by circulating plasma microRNA sequence analysis [48]. Space radiation-induced dysregulated brain inflammation is also reflected in the spleen [40,49], suggesting a robust and whole-body alteration in immune response, which resembles the TBI signature of brain and splenic inflammation [50,51,52]. While still scarce, these reports suggest that radiation-exposed individuals (from uncontrolled sources, therapeutic intervention and space exploration) may be vulnerable to upregulated central and peripheral inflammation, warranting their careful monitoring for TBI-like symptoms and treatment.

## 3. TBI-Induced Stem Cell Dysfunction: Potential of Stem Cell-Based Therapies

### 3.1. The Bone Marrow Niche and Hematopoiesis

**Figure 1:**
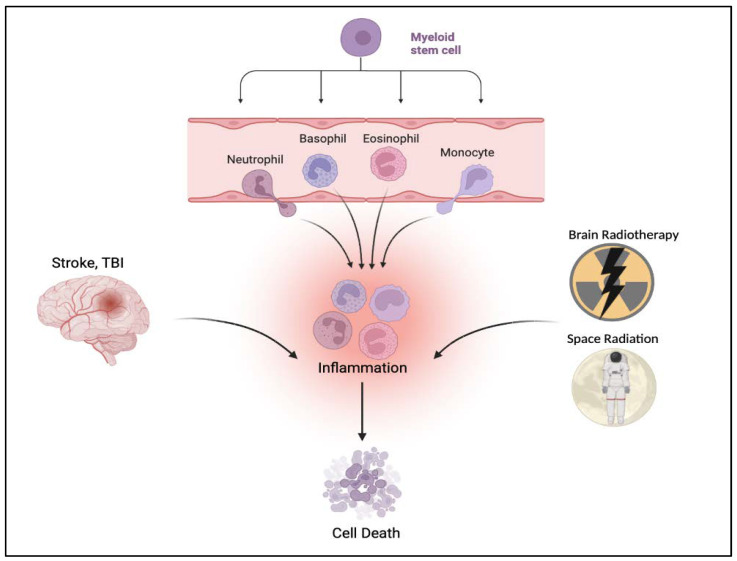
Inflammatory cells, such as neutrophils, dendritic cells and monocytes, arising from myeloid progenitor cells, infiltrate the brain altogether triggering inflammation then cell death in response to neural injury caused by stroke, TBI, brain radiotherapy and space radiation. Figure constructed via Biorender.com.

The bone marrow niche consists of multiple cell types, including hematopoietic stem cells, interacting with extracellular matrix, chemical, and physical factors, altogether forming the microenvironment responsible for maintaining blood cell formation (hematopoiesis) from development to adulthood [53,54]. In healthy normal development and maturation, the bone marrow niche regulates the fate and function of hematopoietic stem cells and their progeny primarily contributing to healthy aging [55]. However, during injury, such as TBI, dysregulation of the bone marrow niche may occur leading to the loss of its pro-survival function [56]. Under this pathologic setting, the bone marrow niche homeostasis is perturbed, switching to a pro-death machinery (Figure 1). Indeed, the injury-induced dysregulation of the bone marrow niche is associated with an inflammatory milieu [57,58]. These documented divergent pro-survival and pro-death functions suggest the double-edged sword role of the bone marrow niche in both homeostatic and inflammatory processes in normal and pathologic conditions, respectively. Notwithstanding, the bone marrow niche has been shown to be involved in the maintenance and dysregulation of hematopoietic stem cells [59] that either facilitate cell survival or cell death. The bone marrow niche may be disrupted by TBI indirectly by signaling molecules in the circulation, suggesting crosstalk between the bone marrow and the injured brain communicating through the inflammatory and lymphatic systems [33,50,51]. Such bone marrow–brain interaction, via the circulation, may confer pro-survival and pro-death occurring, most likely, in both the bloodstream and the brain.

### 3.2. TBI and the Hematopoietic Response

Neurogenic signals are released by the injured brain, which may potentially affect the bone marrow niche or the hematopoietic response to neural injuries [60]. The process of myelopoiesis and the subsequent inflammation in response to injury may propel innate immune cells, such as neutrophils, dendritic cells and monocytes, originating from myeloid progenitor cells, to the site of injury [61,62]. Animal models of ischemic and hemorrhagic stroke detect the occurrence of injury-induced myelopoiesis [63,64]. With some overlapping pathology of brain damage between stroke and TBI, this hematopoietic response to neural injury seen in stroke may also accompany TBI. Indeed, preclinical studies in CNS trauma demonstrate similar myelopoiesis, followed by subsequent inflammation [65,66]. Accumulating evidence also implicates the participation of myelopoiesis and inflammation in space flight and irradiation [67,68]. Taken together, these findings support the notion of a crosstalk between myelopoiesis and inflammation in response to neural injury.

### 3.3. TBI-like Events and Inflammation

The inflammation-ridden pathologic feature of TBI closely approximates the onset and progressive deterioration of debilitating sensory-motor deficits and learning and memory impairments in patients [69]. A closer examination of this inflammatory signature reveals infiltration of inflammatory cells with increased pro-inflammatory cytokines in neurogenic niches in the injured brain [70,71,72]. In parallel, irradiation-induced cognitive impairments are due to disruption of hippocampal neurogenesis [73].

Space flights also alter stem cell adhesion [74], with RNA-sequencing analysis detecting specific genes differentially expressed and associated with mitochondrial metabolism [75]. These new data implicate the major involvement of stem cells in TBI and TBI-like pathology, again highlighting the close association of inflammation with the behavioral and neurostructural manifestations of the disease.

### 3.4. Stem Cell Therapy for Reducing Neural Injury-Induced Inflammation

**Figure 2:**
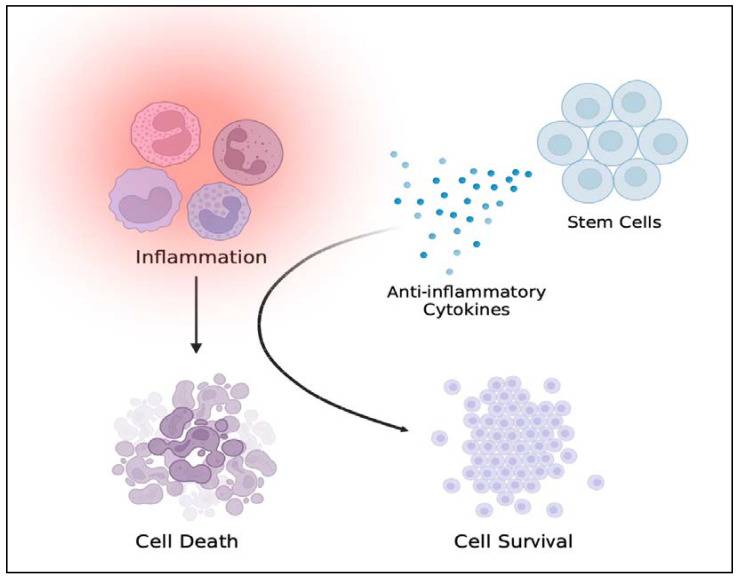
Inflammatory/immune cells are sequestered by stem cells that release anti-inflammatory factors suggesting the potential of stem cell therapy for treating neural injury. Figure constructed via Biorender.com.

To this end, the fact that inflammation plays an exacerbating factor in TBI acts as the impetus for finding a treatment that dampens inflammation to retard the long-term deleterious consequences of TBI. Stem cell therapy as an approach to replenish injured cells, as well as a delivery vehicle for therapeutic molecules, including anti-inflammatory factors, may serve as an appealing TBI treatment (Figure 2). In the laboratory, cell transplantation either directly into the injured brain or peripherally promotes functional recovery in TBI [76,77,78,79,80,81].

Mobilizing resident stem cells, via small molecules, such as granulocyte colony stimulating factor, also affords therapeutic effects in TBI [82,83,84,85]. As noted above, TBI-induced inflammation is rampant in both the brain and the periphery [86,87,88,89]. Accordingly, when contemplating stem cell therapy for TBI, deposition of the grafted stem cells and their secreted factors are most optimal if they hone to the brain as well as to the inflammation-enriched organs, i.e., spleen, to sequester inflammation. In the clinic, stem cell delivery via a lumbar puncture or intravenously reveals safety and modest efficacy in enhancing motor function [90,91,92,93]. Importantly, the improved clinical outcomes coincided with downregulation of inflammatory cytokines IL-1β and IFN-γ [90], which mimicked previously reported preclinical evidence in animal models of TBI [69,70] These findings support the application of stem cell therapy for dampening inflammation and improving motor functions in TBI.

## 4. Conclusions

**Figure 3:**
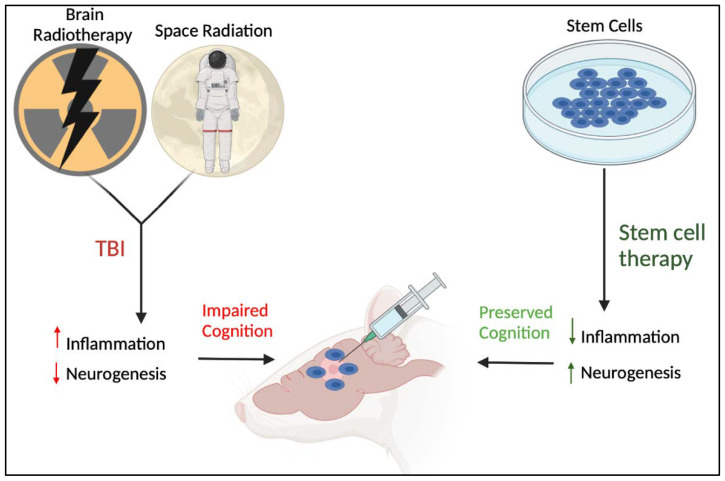
Compelling evidence suggests that brain radiotherapy and space radiation produce TBI-like pathology characterized by aberrant deleterious inflammation and dysfunctional neurogenesis resulting in impaired cognition. Stem cell-based therapies can sequester inflammation and enhance stem cell proliferation in the TBI animals, leading to preserved cognition. Figure constructed via Biorender.com.

TBI remains a significant unmet clinical need with limited therapeutic options. Inflammation, both centrally and peripherally, stands as a main pathologic feature of TBI. Mild TBI and radiation exposure results in inflammation that accompanies the onset and progression of cognitive dysfunctions coincident with stem cell dysregulation (Figure 3). Targeting inflammation-plagued organs with stem cells likely confers therapeutic effects on TBI.

## 5. Future Perspectives 

Neural injury following TBI may evolve into a progressive neurodegenerative disorder. After brain radiotherapy in humans and rodents, and after exposure to space radiation in rodents, pathology and symptoms reminiscent of TBI have been documented. The inflammation in response to brain injury is a key cell death mechanism that can exacerbate the secondary cell death of TBI. Targeting this deleterious inflammation may reduce TBI-induced secondary cell death and its associated functional impairments. To this end, the bone marrow niche stands as a potent microenvironment with a dual action that can either contribute to cell survival or cell death. Recognizing the neurogenic signals and the inflammatory cells may allow a better modulation of the bone marrow niche towards the repair of the injured brain. In particular, the bone marrow is a rich source of stem cells, which can be harvested and transplanted to TBI patients, as well as those diagnosed with similar brain injuries arising from radiation exposure. In the future, elucidating the crosstalk between the inflammatory response to TBI and the bone marrow niche, with special attention to stem cell-based therapy, may prove therapeutically beneficial in our understanding of the pathology and treatment of TBI and TBI-like events.

## Data Availability

Not applicable.

## Abbreviations

TBI	Traumatic brain injury
CSF	Cerebrospinal fluid
CCR2	C-C chemokine receptor 2
CX3CR1	CX3C chemokine receptor 1
GFP	Green fluorescent protein
RFP	Red fluorescent protein
CCL2	Chemokine (C-C motif) ligand 2
C1q	Complement component 1q
C3	Complement component 3
CR3	Complement component R3
RNA	Ribonucleic acid
CNS	Central nervous system
IL-1β	Interleukin-1beta
IFN-γ	Interferon-gamma

## References

[1] Broglio S.P., Eckner J.T., Paulson H.L., Kutcher J.S. (2012). Cognitive Decline and Aging: The role of concussive and subconcussive impacts. Exerc. Sport Sci. Rev..

[2] Cole J.H., Leech R., Sharp D.J., Alzheimer’s Disease Neuroimaging Initiative (2015). Prediction of brain age suggests accelerated atrophy after traumatic brain injury. Ann. Neurol..

[3] Fleminger S., Oliver D.L., Lovestone S., Rabe-Hesketh S., Giora A. (2003). Head injury as a risk factor for Alzheimer’s disease: The evidence 10 years on; A partial replication. J. Neurol. Neurosurg. Psychiatry.

[4] Masel B.E., DeWitt D.S. (2010). Traumatic Brain Injury: A Disease Process, Not an Event. J. Neurotrauma.

[5] Li Y., Li X., Zhang S., Zhao J., Zhu X., Tian G. (2017). Head Injury as a Risk Factor for Dementia and Alzheimer’s Disease: A Systematic Review and Meta-Analysis of 32 Observational Studies. PLoS ONE.

[6] Flanagan S.R. (2015). Invited Commentary on “Centers for Disease Control and Prevention Report to Congress: Traumatic Brain Injury in the United States: Epidemiology and Rehabilitation”. Arch. Phys. Med. Rehabil..

[7] Taylor C.A., Bell J.M., Breiding M.J., Xu L. (2017). Traumatic Brain Injury–Related Emergency Department Visits, Hospitalizations, and Deaths—United States, 2007 and 2013. MMWR Surveill. Summ..

[8] Coronado V.G., Xu L., Basavaraju S.V., McGuire L.C., Wald M.M., Faul M.D., Guzman B.R., Hemphill J.D., Centers for Disease Control and Prevention (CDC) (2002). Surveillance for traumatic brain injury-related deaths-United States, 1997–2007. Morbidity and mortality weekly report. Surveill. Summ..

[9] Coronado V.G., Haileyesus T., Cheng T.A., Bell J.M., Haarbauer-Krupa J., Lionbarger M.R., Flores-Herrera J., McGuire L.C., Gilchrist J. (2015). Trends in Sports- and Recreation-Related Traumatic Brain Injuries Treated in US Emergency Departments: The National Electronic Injury Surveillance System-All Injury Program (NEISS-AIP) 2001-2012. J. Head Trauma Rehabil..

[10] Corrigan J.D., Cuthbert J.P., Harrison-Felix C., Whiteneck G.G., Bell J.M., Miller A.C., Coronado V.G., Pretz C.R. (2014). US Population Estimates of Health and Social Outcomes 5 Years After Rehabilitation for Traumatic Brain Injury. J. Head Trauma Rehabil..

[11] Miller G.F., Daugherty J., Waltzman D., Sarmiento K. (2021). Predictors of traumatic brain injury morbidity and mortality: Examination of data from the national trauma data bank: Predictors of TBI morbidity & mortality. Injury.

[12] Gupte R.P., Brooks W., Vukas R., Pierce J.D., Harris J.L. (2019). Sex Differences in Traumatic Brain Injury: What We Know and What We Should Know. J. Neurotrauma.

[13] Blaya M.O., Raval A.P., Bramlett H.M. (2022). Traumatic brain injury in women across lifespan. Neurobiol. Dis..

[14] Malec J.F., Brown A.W., Leibson C.L., Flaada J.T., Mandrekar J.N., Diehl N.N., Perkins P.K. (2007). The Mayo Classification System for Traumatic Brain Injury Severity. J. Neurotrauma.

[15] Dijkland S.A., Foks K.A., Polinder S., Dippel D.W., Maas A.I., Lingsma H.F., Steyerberg E.W. (2020). Prognosis in Moderate and Severe Traumatic Brain Injury: A Systematic Review of Contemporary Models and Validation Studies. J. Neurotrauma.

[16] Galgano M., Toshkezi G., Qiu X., Russell T., Chin L., Zhao L.-R. (2017). Traumatic Brain Injury: Current Treatment Strategies and Future Endeavors. Cell Transplant..

[17] Faden A.I., Loane D. (2015). Chronic Neurodegeneration After Traumatic Brain Injury: Alzheimer Disease, Chronic Traumatic Encephalopathy, or Persistent Neuroinflammation?. Neurotherapeutics.

[18] Washington P.M., Villapol S., Burns M.P. (2016). Polypathology and dementia after brain trauma: Does brain injury trigger distinct neurodegenerative diseases, or should they be classified together as traumatic encephalopathy?. Exp. Neurol..

[19] Sullivan D.R., Miller M.W., Wolf E.J., Logue M.W., Robinson M.E., Fortier C.B., Fonda J.R., Wang D., Milberg W.P., McGlinchey R.E. (2020). Cerebral perfusion is associated with blast exposure in military personnel without moderate or severe TBI. J. Cereb. Blood Flow Metab..

[20] Wright W.G., Handy J.D., Haskell M.A., Servatius M.L., Servatius R.J. (2022). History of Mild Traumatic Brain Injury Affects Static Balance under Complex Multisensory Manipulations. J. Neurotrauma.

[21] Lippa S.M., French L.M., Brickell T.A., Driscoll M.A., Glazer M.E., Tippett C.E., Sullivan M.J., Lange R.T. (2021). Post-Traumatic Stress Disorder Symptoms Are Related to Cognition after Complicated Mild and Moderate Traumatic Brain Injury but Not Severe and Penetrating Traumatic Brain Injury. J. Neurotrauma.

[22] Corrigan F., Cernak I., McAteer K., Hellewell S.C., Rosenfeld J.V., Turner R.J., Vink R. (2021). NK1 antagonists attenuate tau phosphorylation after blast and repeated concussive injury. Sci. Rep..

[23] Morin A., Mouzon B., Ferguson S., Paris D., Saltiel N., Lungmus C., Mullan M., Crawford F. (2018). Treatment with Nilvadipine Mitigates Inflammatory Pathology and Improves Spatial Memory in Aged hTau Mice After Repetitive Mild TBI. Front. Aging Neurosci..

[24] Jurick S.M., Crocker L.D., Merritt V.C., Sanderson-Cimino M.E., Keller A.V., Glassman L.H., Twamley E.W., Rodgers C.S., Schiehser D.M., Aupperle R.L. (2021). Independent and Synergistic Associations Between TBI Characteristics and PTSD Symptom Clusters on Cognitive Performance and Postconcussive Symptoms in Iraq and Afghanistan Veterans. J. Neuropsychiatry Clin. Neurosci..

[25] Verboon L.N., Patel H.C., Greenhalgh A.D. (2021). The Immune System’s Role in the Consequences of Mild Traumatic Brain Injury (Concussion). Front. Immunol..

[26] Chaban V., Clarke G.J., Skandsen T., Islam R., Einarsen C.E., Vik A., Damås J.K., Mollnes T.E., Håberg A.K., Pischke S.E. (2020). Systemic Inflammation Persists the First Year after Mild Traumatic Brain Injury: Results from the Prospective Trondheim Mild Traumatic Brain Injury Study. J. Neurotrauma.

[27] Gill J., Latour L., Diaz-Arrastia R., Motamedi V., Turtzo C., Shahim P., Mondello S., DeVoto C., Veras E., Hanlon D. (2018). Glial fibrillary acidic protein elevations relate to neuroimaging abnormalities after mild TBI. Neurology.

[28] Vedantam A., Brennan J., Levin H.S., McCarthy J.J., Dash P.K., Redell J.B., Yamal J.-M., Robertson C.S. (2021). Early versus Late Profiles of Inflammatory Cytokines after Mild Traumatic Brain Injury and Their Association with Neuropsychological Outcomes. J. Neurotrauma.

[29] Molina I.S.M., Salo R.A., Abdollahzadeh A., Tohka J., Gröhn O., Sierra A. (2020). *In Vivo* Diffusion Tensor Imaging in Acute and Subacute Phases of Mild Traumatic Brain Injury in Rats. Eneuro.

[30] Weiss E., Dhir T., Collett A., Reola M., Kaplan M., Minimo C., Omert L., Leung P. (2020). Effect of complement C1-esterase inhibitor on brain edema and inflammation after mild traumatic brain injury in an animal model. Clin. Exp. Emerg. Med..

[31] Tweedie D., Karnati H.K., Mullins R., Pick C.G., Hoffer B.J., Goetzl E.J., Kapogiannis D., Greig N.H. (2020). Time-dependent cytokine and chemokine changes in mouse cerebral cortex following a mild traumatic brain injury. ELife.

[32] Xu M.L., Yue J.K., Korley F.K., Puccio A.M., Yuh E.L., Sun M.X., Rabinowitz M., Vassar M.M., Taylor S.R., Winkler E.A. (2021). High-Sensitivity C-Reactive Protein is a Prognostic Biomarker of Six-Month Disability after Traumatic Brain Injury: Results from the TRACK-TBI Study. J. Neurotrauma.

[33] Glushakova O.Y., Glushakov A.O., Borlongan C.V., Valadka A.B., Hayes R.L., Glushakov A.V. (2018). Role of Caspase-3-Mediated Apoptosis in Chronic Caspase-3-Cleaved Tau Accumulation and Blood–Brain Barrier Damage in the Corpus Callosum after Traumatic Brain Injury in Rats. J. Neurotrauma.

[34] Shahim P., Politis A., van der Merwe A., Moore B., Ekanayake V., Lippa S.M., Chou Y.-Y., Pham D.L., Butman J.A., Diaz-Arrastia R. (2020). Time course and diagnostic utility of NfL, tau, GFAP, and UCH-L1 in subacute and chronic TBI. Neurology.

[35] Morganti J.M., Jopson T.D., Liu S., Riparip L.-K., Guandique C.K., Gupta N., Ferguson A., Rosi S. (2015). CCR2 Antagonism Alters Brain Macrophage Polarization and Ameliorates Cognitive Dysfunction Induced by Traumatic Brain Injury. J. Neurosci..

[36] Morganti J.M., Riparip L.-K., Rosi S. (2016). Call Off the Dog(ma): M1/M2 Polarization Is Concurrent following Traumatic Brain Injury. PLoS ONE.

[37] Morganti J.M., Riparip L.-K., Chou A., Liu S., Gupta N., Rosi S. (2016). Age exacerbates the CCR2/5-mediated neuroinflammatory response to traumatic brain injury. J. Neuroinflammation.

[38] Chou A., Krukowski K., Morganti J.M., Riparip L.-K., Rosi S. (2018). Persistent Infiltration and Impaired Response of Peripherally-Derived Monocytes after Traumatic Brain Injury in the Aged Brain. Int. J. Mol. Sci..

[39] Krukowski K., Chou A., Feng X., Tiret B., Paladini M.-S., Riparip L.-K., Chaumeil M.M., Lemere C., Rosi S. (2018). Traumatic Brain Injury in Aged Mice Induces Chronic Microglia Activation, Synapse Loss, and Complement-Dependent Memory Deficits. Int. J. Mol. Sci..

[40] Allen A.R., Eilertson K., Chakraborti A., Sharma S., Baure J., Habdank-Kolaczkowski J., Allen B., Rosi S., Raber J., Fike J.R. (2013). Radiation exposure prior to traumatic brain injury induces responses that differ as a function of animal age. Int. J. Radiat. Biol..

[41] Feng X., Frias E.S., Paladini M.S., Chen D., Boosalis Z., Becker M., Gupta S., Liu S., Gupta N., Rosi S. (2021). Functional role of brain-engrafted macrophages against brain injuries. J. Neuroinflammation.

[42] Feng X., Jopson T.D., Paladini M.S., Liu S., West B.L., Gupta N., Rosi S. (2016). Colony-stimulating factor 1 receptor blockade prevents fractionated whole-brain irradiation-induced memory deficits. J. Neuroinflammation.

[43] Feng X., Liu S., Chen D., Rosi S., Gupta N. (2018). Rescue of cognitive function following fractionated brain irradiation in a novel preclinical glioma model. ELife.

[44] Belarbi K., Jopson T., Arellano C., Fike J.R., Rosi S. (2013). CCR2 Deficiency Prevents Neuronal Dysfunction and Cognitive Impairments Induced by Cranial Irradiation. Cancer Res..

[45] Hohsfield L.A., Najafi A.R., Ghorbanian Y., Soni N., Hingco E.E., Kim S.J., Jue A.D., Swarup V., Inlay M.A., Green K.N. (2020). Effects of long-term and brain-wide colonization of peripheral bone marrow-derived myeloid cells in the CNS. J. Neuroinflammation.

[46] Krukowski K., Grue K., Becker M., Elizarraras E., Frias E.S., Halvorsen A., Koenig-Zanoff M., Frattini V., Nimmagadda H., Feng X. (2021). The impact of deep space radiation on cognitive performance: From biological sex to biomarkers to countermeasures. Sci. Adv..

[47] Krukowski K., Grue K., Frias E.S., Pietrykowski J., Jones T., Nelson G., Rosi S. (2018). Female mice are protected from space radiation-induced maladaptive responses. Brain Behav. Immun..

[48] Paul A.M., Cheng-Campbell M., Blaber E.A., Anand S., Bhattacharya S., Zwart S.R., Crucian B.E., Smith S.M., Meller R., Grabham P. (2020). Beyond Low-Earth Orbit: Characterizing Immune and microRNA Differentials following Simulated Deep Spaceflight Conditions in Mice. iScience.

[49] Laiakis E.C., Shuryak I., Deziel A., Wang Y.W., Barnette B.L., Yu Y., Ullrich R.L., Fornace A.J., Emmett M.R. (2021). Effects of low dose space radiation exposures on the splenic metabolome. Int. J. Mol. Sci..

[50] Lee J.-Y., Acosta S., Tuazon J.P., Xu K., Nguyen H., Lippert T., Liska M.G., Semechkin A., Garitaonandia I., Gonzalez R. (2019). Human parthenogenetic neural stem cell grafts promote multiple regenerative processes in a traumatic brain injury model. Theranostics.

[51] Marcet P., Santos N., Borlongan C.V. (2017). When friend turns foe: Central and peripheral neuroinflammation in central nervous system injury. Neuroimmunol. Neuroinflammation.

[52] Toyoshima A., Yasuhara T., Kameda M., Morimoto J., Takeuchi H., Wang F., Sasaki T., Sasada S., Shinko A., Wakamori T. (2015). Intra-Arterial Transplantation of Allogeneic Mesenchymal Stem Cells Mounts Neuroprotective Effects in a Transient Ischemic Stroke Model in Rats: Analyses of Therapeutic Time Window and Its Mechanisms. PLoS ONE.

[53] Amaroli A., Pasquale C., Zekiy A., Benedicenti S., Marchegiani A., Sabbieti M.G., Agas D. (2022). Steering the multipotent mesenchymal cells towards an anti-inflammatory and osteogenic bias via photobiomodulation therapy: How to kill two birds with one stone. J. Tissue Eng..

[54] Granata V., Crisafulli L., Nastasi C., Ficara F., Sobacchi C. (2022). Bone Marrow Niches and Tumour Cells: Lights and Shadows of a Mutual Relationship. Front. Immunol..

[55] Morrison S.J., Scadden D.T. (2014). The bone marrow niche for haematopoietic stem cells. Nature.

[56] Fröbel J., Landspersky T., Percin G., Schreck C., Rahmig S., Ori A., Nowak D., Essers M., Waskow C., Oostendorp R.A.J. (2021). The Hematopoietic Bone Marrow Niche Ecosystem. Front. Cell Dev. Biol..

[57] Bartl M., Xylaki M., Bähr M., Weber S., Trenkwalder C., Mollenhauer B. (2022). Evidence for immune system alterations in peripheral biological fluids in Parkinson’s disease. Neurobiol. Dis..

[58] Hasselbalch H.C. (2013). Chronic inflammation as a promotor of mutagenesis in essential thrombocythemia, polycythemia vera and myelofibrosis. A human inflammation model for cancer development?. Leuk. Res..

[59] Waclawiczek A., Hamilton A., Rouault-Pierre K., Abarrategi A., Garcia-Albornoz M., Miraki-Moud F., Bah N., Gribben J., FitzGibbon J., Taussig D. (2020). Mesenchymal niche remodeling impairs hematopoiesis via stanniocalcin 1 in acute myeloid leukemia. J. Clin. Investig..

[60] Girard D., Torossian F., Oberlin E., Alexander K.A., Gueguen J., Tseng H.-W., Genêt F., Lataillade J.-J., Salga M., Levesque J.-P. (2021). Neurogenic Heterotopic Ossifications Recapitulate Hematopoietic Stem Cell Niche Development Within an Adult Osteogenic Muscle Environment. Front. Cell Dev. Biol..

[61] Foertsch S., Reber S.O. (2020). The role of physical trauma in social stress-induced immune activation. Neurosci. Biobehav. Rev..

[62] Hawthorne A.L., Popovich P.G. (2011). Emerging Concepts in Myeloid Cell Biology after Spinal Cord Injury. Neurotherapeutics.

[63] Fang M.M., Barman P.K., Thiruppathi M., Mirza R.E., McKinney R.D., Deng J., Christman J.W., Du X., Fukai T., Ennis W.J. (2018). Oxidant Signaling Mediated by Nox2 in Neutrophils Promotes Regenerative Myelopoiesis and Tissue Recovery following Ischemic Damage. J. Immunol..

[64] Wolf D., Ley K. (2015). Waking Up the Stem Cell Niche: How hematopoietic stem cells generate inflammatory monocytes after stroke. Circ. Res..

[65] Mazzitelli J.A., Smyth L.C.D., Cross K.A., Dykstra T., Sun J., Du S., Mamuladze T., Smirnov I., Rustenhoven J., Kipnis J. (2022). Cerebrospinal fluid regulates skull bone marrow niches via direct access through dural channels. Nat. Neurosci..

[66] Shi S.X., Shi K., Liu Q. (2021). Brain injury instructs bone marrow cellular lineage destination to reduce neuroinflammation. Sci. Transl. Med..

[67] Huin-Schohn C., Guéguinou N., Schenten V., Bascove M., Koch G.G., Baatout S., Tschirhart E., Frippiat J. (2012). Gravity changes during animal development affect IgM heavy-chain transcription and probably lymphopoiesis. FASEB J..

[68] Rostami T., Mousavi S.A., Kiumarsi A., Kasaeian A., Rad S., Yaghmaie M., Ghavamzadeh A., Mousavi S.A. (2022). Radiation-free reduced-intensity hematopoietic stem cell transplantation with in vivo T-cell depletion from matched related and unrelated donors for Fanconi anemia: Prognostic factor analysis. Exp. Hematol..

[69] Acosta S.A., Tajiri N., Shinozuka K., Ishikawa H., Sanberg P.R., Sanchez-Ramos J., Song S., Kaneko Y., Borlongan C.V. (2014). Combination Therapy of Human Umbilical Cord Blood Cells and Granulocyte Colony Stimulating Factor Reduces Histopathological and Motor Impairments in an Experimental Model of Chronic Traumatic Brain Injury. PLoS ONE.

[70] Acosta S.A., Tajiri N., Shinozuka K., Ishikawa H., Grimmig B., Diamond D., Sanberg P.R., Bickford P., Kaneko Y., Borlongan C.V. (2013). Long-Term Upregulation of Inflammation and Suppression of Cell Proliferation in the Brain of Adult Rats Exposed to Traumatic Brain Injury Using the Controlled Cortical Impact Model. PLoS ONE.

[71] Lozano D., Gonzales-Portillo G.S., Acosta S., de la Pena I., Tajiri N., Kaneko Y., Borlongan C.V. (2015). Neuroinflammatory responses to traumatic brain injury: Etiology, clinical consequences, and therapeutic opportunities. Neuropsychiatr. Dis. Treat..

[72] Pabón M.M., Acosta S., Guedes V.A., Tajiri N., Kaneko Y., Borlongan C.V. (2016). Brain Region-Specific Histopathological Effects of Varying Trajectories of Controlled Cortical Impact Injury Model of Traumatic Brain Injury. CNS Neurosci. Ther..

[73] Rola R., Fishman K., Baure J., Rosi S., Lamborn K.R., Obenaus A., Nelson G.A., Fike J.R. (2008). Hippocampal Neurogenesis and Neuroinflammation after Cranial Irradiation with ^56^Fe Particles. Radiat. Res..

[74] Lin X., Zhang K., Wei D., Tian Y., Gao Y., Chen Z., Qian A. (2020). The Impact of Spaceflight and Simulated Microgravity on Cell Adhesion. Int. J. Mol. Sci..

[75] Wnorowski A., Sharma A., Chen H., Wu H., Shao N.-Y., Sayed N., Liu C., Countryman S., Stodieck L.S., Rubins K.H. (2019). Effects of Spaceflight on Human Induced Pluripotent Stem Cell-Derived Cardiomyocyte Structure and Function. Stem Cell Rep..

[76] Cozene B., Sadanandan N., Farooq J., Kingsbury C., Park Y.J., Wang Z.-J., Moscatello A., Saft M., Cho J., Gonzales-Portillo B. (2021). Mesenchymal Stem Cell-Induced Anti-Neuroinflammation Against Traumatic Brain Injury. Cell Transplant..

[77] Lee S., Mattingly A., Lin A., Sacramento J., Mannent L., Castel M.-N., Canolle B., Delbary-Gossart S., Ferzaz B., Morganti J.M. (2016). A novel antagonist of p75NTR reduces peripheral expansion and CNS trafficking of pro-inflammatory monocytes and spares function after traumatic brain injury. J. Neuroinflammation.

[78] Neal E., Liska M.G., Lippert T., Lin R., Gonzalez M., Russo E., Xu K., Ji X., Vale F.L., Van Loveren H. (2018). An update on intracerebral stem cell grafts. Expert Rev. Neurother..

[79] Gao J., Grill R.J., Dunn T.J., Bedi S., Labastida J.A., Hetz R.A., Xue H., Thonhoff J.R., DeWitt D.S., Prough D.S. (2016). Human Neural Stem Cell Transplantation-Mediated Alteration of Microglial/Macrophage Phenotypes after Traumatic Brain Injury. Cell Transplant..

[80] Barretto M.T., Park E., Telliyan M.T., Liu E., Gallagher D., Librach C., Baker A. (2021). Vascular Dysfunction after Modeled Traumatic Brain Injury Is Preserved with Administration of Umbilical Cord Derived Mesenchymal Stromal Cells and Is Associated with Modulation of the Angiogenic Response. J. Neurotrauma.

[81] Caplan H.W., Prabhakara K.S., Furman N.E.T., Zorofchian S., Kumar A., Martin C., Xue H., Olson S.D., Cox C.S. (2021). Combination therapy with Treg and mesenchymal stromal cells enhances potency and attenuation of inflammation after traumatic brain injury compared to monotherapy. Stem Cells.

[82] Peña I.D., Borlongan C.V. (2015). Translating G-CSF as an Adjunct Therapy to Stem Cell Transplantation for Stroke. Transl. Stroke Res..

[83] Song S., Kong X., Borlongan C., Sava V., Sanchez-Ramos J. (2021). Granulocyte Colony-Stimulating Factor Enhances Brain Repair Following Traumatic Brain Injury Without Requiring Activation of Cannabinoid Receptors. Cannabis Cannabinoid Res..

[84] Qiu X., Ping S., Kyle M., Chin L., Zhao L.-R. (2020). Long-term beneficial effects of hematopoietic growth factors on brain repair in the chronic phase of severe traumatic brain injury. Exp. Neurol..

[85] Song S., Kong X., Acosta S., Sava V., Borlongan C., Sanchez-Ramos J. (2016). Granulocyte colony-stimulating factor promotes behavioral recovery in a mouse model of traumatic brain injury. J. Neurosci. Res..

[86] Zhao Y., Mu H., Huang Y., Li S., Wang Y., Stetler R.A., Bennett M., Dixon C.E., Chen J., Shi Y. (2022). Microglia-specific deletion of histone deacetylase 3 promotes inflammation resolution, white matter integrity, and functional recovery in a mouse model of traumatic brain injury. J. Neuroinflammation.

[87] Lippert T., Borlongan C.V. (2019). Prophylactic treatment of hyperbaric oxygen treatment mitigates inflammatory response via mitochondria transfer. CNS Neurosci. Ther..

[88] Mason H.D., Johnson A.M., Mihelson N.A., Mastorakos P., McGavern D.B. (2021). Glia limitans superficialis oxidation and breakdown promote cortical cell death after repetitive head injury. JCI Insight.

[89] Newell-Rogers M.K., Rogers S.K., Tobin R.P., Mukherjee S., Shapiro L.A. (2020). Antagonism of Macrophage Migration Inhibitory Factory (MIF) after Traumatic Brain Injury Ameliorates Astrocytosis and Peripheral Lymphocyte Activation and Expansion. Int. J. Mol. Sci..

[90] Tian C., Wang X., Wang X., Wang L., Wang X., Wu S., Wan Z. (2013). Autologous Bone Marrow Mesenchymal Stem Cell Therapy in the Subacute Stage of Traumatic Brain Injury by Lumbar Puncture. Exp. Clin. Transplant..

[91] Cox C.S., Hetz R.A., Liao G.P., Aertker B.M., Ewing-Cobbs L., Juranek J., Savitz S.I., Jackson M.L., Romanowska-Pawliczek A.M., Triolo F. (2017). Treatment of Severe Adult Traumatic Brain Injury Using Bone Marrow Mononuclear Cells. Stem Cells.

[92] Wang J., Wang J., Li X., Shu K. (2022). Cell-Derived Exosomes as Therapeutic Strategies and Exosome-Derived microRNAs as Biomarkers for Traumatic Brain Injury. J. Clin. Med..

[93] Alizada M., Lin S., Gao H. (2021). Recent advances in the treatment of traumatic brain injury with autologous and non-autologous multipotent stem and progenitor cells: Preclinical models and clinical trials. Folia Neuropathol..

